# Uptake of substances into living mammalian cells by microwave induced perturbation of the plasma membrane

**DOI:** 10.1038/s41598-024-71401-7

**Published:** 2024-09-06

**Authors:** Manuela Milden-Appel, Markus Paravicini, Jannick P. Milden, Martin Schüßler, Rolf Jakoby, M. Cristina Cardoso

**Affiliations:** 1 Cell Biology and Epigenetics, Department of Biology, Technical University of Darmstadt, Darmstadt, Germany; 2 Institute of Microwave Engineering and Photonics, Technical University of Darmstadt, Darmstadt, Germany

**Keywords:** Cellular uptake, Cell viability, Fluorescence microscopy, Mammalian cells, Microwave-induced poration, Biological techniques, Cell biology

## Abstract

Delivering foreign molecules and genetic material into cells is a crucial process in life sciences and biotechnology, resulting in great interest in effective cell transfection methods. Importantly, physical transfection methods allow delivery of molecules of different chemical composition and are, thus, very flexible. Here, we investigated the influence of microwave radiation on the transfection and survival of mammalian cells. We made use of an optimized microwave-poration device and analyzed its performance (frequency and electric field strength) in comparison with simulations. We, then, tested the effect of microwave irradiation on cells and found that 18 GHz had the least impact on cell survival, viability, cell division and genotoxicity while 10 GHz drastically impacted cell physiology. Using live-cell fluorescence microscopy and image analysis, we tested the uptake of small chemical substances, which was most efficient at 18 GHz and correlated with electric field strength and frequency. Finally, we were able to obtain cellular uptake of molecules of very different chemical composition and sizes up to whole immunoglobulin antibodies. In conclusion, microwave-induced poration enables the uptake of widely different substances directly into mammalian cells growing as adherent cultures and with low physiological impact.

## Introduction

In modern biotechnology and life sciences research, delivering foreign molecules or genetic material into cells is a crucial process, unlocking new possibilities for studying cellular function and developing advanced therapies. Various methods to enable cellular uptake of substances exist, including physical techniques like electroporation, chemical methods using cationic lipids or biological approaches making use of virus infection. However, each of these methods has its limitations. While the physical methods in principle allow the cellular uptake of any substance type, the other methods are more limited to the type of substances that can be delivered. Nonetheless, physical methods like electroporation require that cells are put into suspension, usually by treating them with trypsin allowing the cells to detach before being subjected to the electrical field and then allowed to adhere again to the substrate, which in general takes several hours. This subjects the cells to an enzymatic treatment and interrupts the observation of the cells until they attach again coupled with a relatively high level of toxicity and cell death. In classical electroporation, pulses with a frequency range from kHz to MHz and with high electric field strengths of up to 100,000 V cm^-1^ and duration time from ps to ms^1–3^ are normally used. Although the method works for many cells and substances to be delivered, a high cell death is observed due to the high electrical field strength^4^.

Microwave (MW) treatment is a physical technique, which utilizes electromagnetic waves to induce the cellular uptake of molecules^5–7^. Microwave-induced cell membrane permeabilization is a transfection method, which is non-toxic and not limited to only a few substances. Furthermore, the method is a more gentle technique operating in the GHz range, because it requires only electrical field strengths of up to 50–200 V cm^-1^ to achieve uptake of substances^5,8^. Studies have been conducted to examine the impact of MW irradiation on the cell membrane permeability of microorganisms. Over the past 15 years, several groups^8–11^ have reported an increase in cell membrane permeability in various bacteria and yeast upon MW irradiation. Only a few studies have been performed using human cells. In one study in human suspension cells (red blood cells), Nguyen et al.^12^ investigated the impact of high-frequency MW irradiation at 18 GHz on the uptake of two types of fluorescent silica nanospheres (23.5 nm and 46.3 nm in diameter). The results showed that MW irradiation significantly enhanced nanosphere uptake compared to the control group, which received no irradiation at the same temperature, and the original cell morphology remained unchanged. In another study conducted by Mazinani et al.^6^, it was found that exposing mammalian MCF-7 breast and PC-3 prostate cancer cells (both growing as adherent cell cultures) to 10 W MW irradiation at 2.45 GHz significantly increased their uptake of the anti-cancer drug doxorubicin. This heightened doxorubicin uptake was associated with a notable increase in doxorubicin induced cell death, whereas cells treated solely with microwaves did not exhibit an elevated mortality rate. Perera et al.^7^ reported that exposure to 18 GHz induced transient cell membrane permeabilization in PC12 human pheochromocytoma cells. Approximately 90% of the cells exhibited permeability for 9 min. This permeability was confirmed by the uptake of silica nanospheres (with a diameter of approximately 23.5 nm). The authors proposed that electromagnetic field (EMF) radiation could cause membrane depolarization and transient cell permeability without affecting cell viability. In a second study, Perera et al.^13^ utilized silica core–shell gold nanospheres with a diameter of 20 ± 5 nm to explore the localization of nanospheres in PC 12 cells after high-frequency electromagnetic fields (HF-EMF) exposure. The internalization of the particles was confirmed using fluorescence microscopy and transmission electron microscopy and it was proposed to involve both active and passive translocation mechanisms. In an in vitro study, Perera et al.^14^ showed evidence of silica nanospheres moving through giant unilamellar vesicles (GUVs) without damaging their integrity after exposure to 18 GHz HF-EMF. It is noteworthy that exposing cells to 18 GHz EMF was reported to have no observable detrimental effects on cell morphology, attachment, proliferation, or differentiation^13^.

In our previous work^5^, we were able to show the uptake of the red labeled peptide LGQQQPFPPQQL-5TAMRA (Pep-5T) in adherent mammalian cells with a novel MW electroporation system. Our prototype allowed monitoring the MW-induced uptake process kinetics at 18 GHz with live cell time lapse confocal microscopy. Hence, this device was suitable to culture, manipulate and observe cells over several days. Subsequently, we were also able to show cellular uptake of the anti-H2AX specific nanobody in adherent mammalian cells after MW-induced electroporation at 10 GHz and 0.7 W for 15 min with a new prototype design^15^.

Here, we introduce a modified version of the MW porator (MWP) device from^15^ and investigate the cellular viability to microwave treatment as well as the MW-induced cellular uptake of substances in human and mouse cells. In particular, we analyze the most important influencing parameters: frequency, electrical field strength, temperature and different substances. The device was designed to operate in a frequency range from 0.5 to 18 GHz and to allow the analysis of the cells on a microscope. With the resulting data, a relation between the electrical field strength and the frequency on the rise of the cell membrane permeabilization was developed. We present an overview of the design of the MWP device, followed by the comparison of simulation and measurement data for the S-parameters, which are ratios of the incident and reflected or transmitted and reflected waves giving an impression of the effective power consumption of the MWP device. The simulated and measured S-parameters were used to compute the electrical field strength inside the MWP chamber. Using this device with adherent mammalian cells we measured cell survival rates at different frequencies, electrical field strengths and temperatures. In addition, we measured the effects of MW irradiation at different frequencies on cell viability, cell division and genotoxicity. Next, the cellular uptake rates of small fluorescent dyes in human cells for different frequencies, electrical field strengths and application times are shown and analyzed. Finally, we extended the uptake experiments to mouse cells and to different substances, including whole immunoglobulin antibodies.

## Methods

### Design of the microwave porator device

**Fig. 1:**
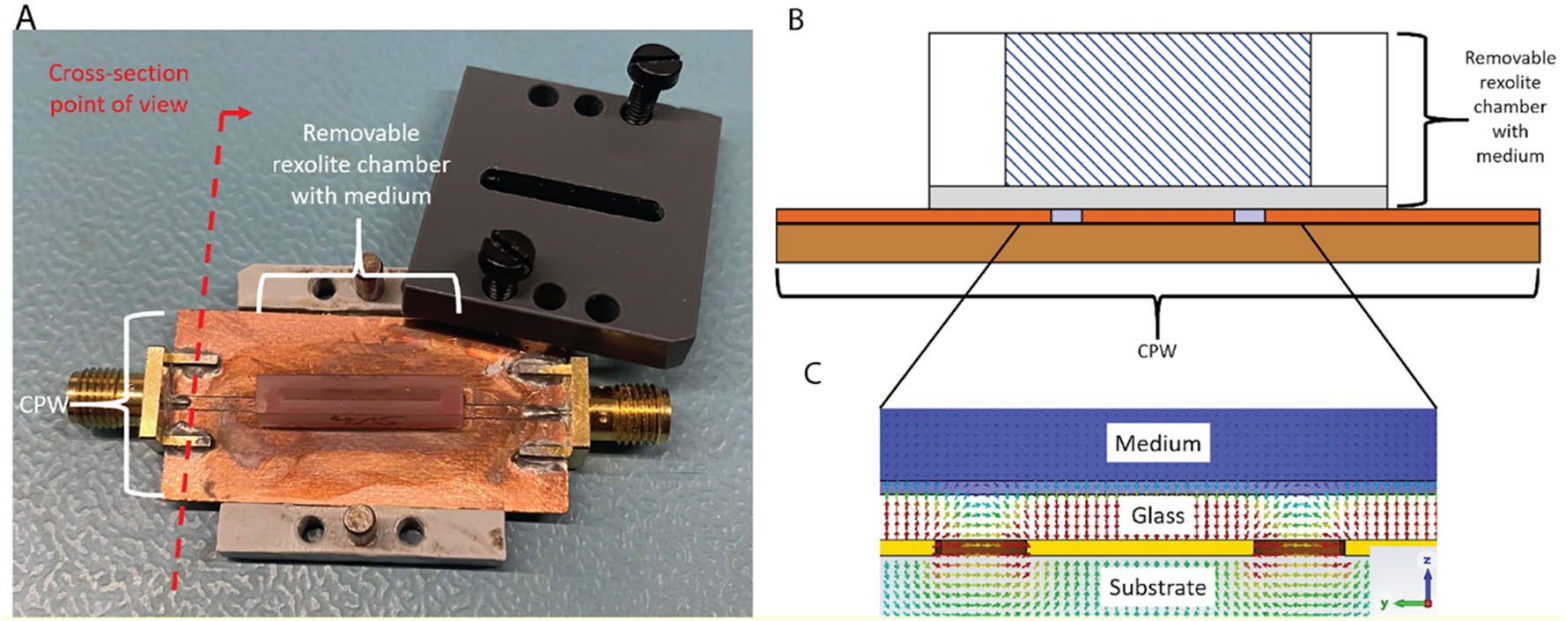
Design of the microwave porator device. (**A**) An image of the fabricated microwave porator (MWP) device with a lid (black part) to fix the position of the MWP chamber on the coplanar waveguide (CPW). (**B**) Cross-section view of the MWP device showing the setup of the chamber and the MWP device. (**C**) Electrical field distribution along the MWP device showing TE_020_ operating mode in the MWP chamber.

To analyze the influence of the microwave radiation on different cell lines, a MWP device was used, which is the further development of the concept shown in Paravicini et al.^15^. The main part of the MWP device is based on a coplanar waveguide (CPW), which was fabricated on a Rogers RT duroid 5880 substrate. Onto the CPW, a removable rexolite (1422, C-Lec Plastics) container with a glued glass bottom (AF 32 eco from Schott AG) with a glass thickness of 100 μm was placed. Rexolite 1422 is a cross-linked polystyrene for microwave applications. The material is resistant to many chemicals, has low electrical losses and the dielectric constant is constant up to a frequency of 500 GHz. The rexolite 1422 container was designed to be removable to reach high experimental throughput and to allow better cleaning of the chamber compared to a previous device^5^. To avoid air gaps between the glass and the CPW and to hold the chamber in its position, a customized lid was added on top of the device, which can be screwed with the device, adding contact pressure. An image of the device as well as a cross-section is shown in Fig. 1A + B. The MWP device itself is designed to operate in a frequency range from 0.5 MHz to 18 GHz, which allows the analysis of the influence of a broad frequency range as well as different electrical field strengths. Due to its design, the transversal electrical (TE_020_) mode is propagating, which results in the guidance of the MW inside the chamber along the line of the CPW and not, as usually nearby a CPW, along the gaps. This results in a magnetic field along the propagation direction of the wave. This is shown in Fig. 1C. Due to the electrical field distribution inside the chamber, where the electrical field strength is high at the input port of the chamber and is decreasing over the length of the chamber, the influence of the electrical field strength on the uptake process of different substances can be analyzed.

### Cells

All cells used were tested for mycoplasma and deemed free of contamination at the time of use. Experiments were performed using human cervix carcinoma HeLa Kyoto^16^ or mouse C2C12^17^ myoblasts cells. Authentication of HeLa cells was performed using short tandem repeat analysis (ATCC, Manassas, Virginia, USA). Mouse myoblasts were authenticated by the ability to differentiate into skeletal muscle and the typical mouse heterochromatin spatial distribution using DNA staining with 4,6-diamidino-2-phenylindole (DAPI, 1 g mL^−1^, cat# D9542, Sigma-Aldrich, St Louis, MO, USA). The cells were cultured in Dulbecco's Modified Eagle's Medium (DMEM Cat. No.: 41965039 Gibco, Paisley, United Kingdom) supplemented with 10 or 20% fetal calf serum for human and mouse cells, respectively (Cat. No.: FBS-11a, Capricorn Scientific), 220 mM glutamine (Cat. No.: M11-04, Sigma Aldrich Chemie GmbH (Merck)), 50 μg mL^−1^ gentamicin (Cat.No.: p11-005, Sigma-Aldrich Chemie GmbH (Merck)) and 100 mM sodium pyruvate (Cat.No.: S8683, Sigma Aldrich Chemie GmbH (Merck)) at 37 °C and 5% CO_2_.

### Cell microwave poration

One day before usage, 4000–5000 cells in total were seeded onto the gelatine-coated glass (Cat. Nr.: G2500-100G, Sigma-Aldrich Chemie GmbH (Merck)) of the chamber of the MWP device, to achieve a homogeneous confluence of 50–60% on the next day. Before the MW treatment, the media was removed and 100 μL of 20 mM HEPES buffer (Cat.No.: 7365-45-9, Sigma-Aldrich Chemie GmbH (Merck)) buffered with KOH to a pH of 7.4 was added including or not the substances listed in Table S1. For the microwave treatment, the chamber was fixed onto the device using a plastic lid and screws to ensure uniform positioning of the chambers in all experiments. The microwave experimental setup consisted of a power source (model MG3692A, Anritsu), a RF power amplifier (model BLMA 0818-20D, Bonn Elektronik GmbH), a power meter (model NRV 828.2511.02, Rohde & Schwarz) as well as a microwave transfection device shown in Fig. 1A, constructed using Rogers RT/duroid 5880 substrate, similar to the one in Ref.^15^. The chambers were constructed out of rexolite and a 100 μm thin glass (AF32 eco, Schott) as chamber bottom as mentioned above, glued with silicone glue SCRINTEC® (2024.1, Carl Roth GmbH + Co. KG). To monitor the temperatures during experiments, a thermal controller (model 2116, Eurotherm) with a thermocouple element (Type K, model R416.110.00, D/C: A06.37, Radiall) and a fiber glass thermometer (OEM-PLUS, Weidmann Technologies GmbH) with a TS5 sensor were used (see Table S2).

### Immunofluorescence

For cell genotoxic analysis, HeLa Kyoto cells were fixed in ice cold methanol for 10 min, followed by blocking in 1% BSA/PBS (bovine serum albumin in 1 × phosphate-buffered saline) for 20 min. The primary antibody anti histone H2AX phosphoSer139 (Cat.No.: 05-636, Sigma-Aldrich Chemie GmbH (Merck)) was diluted 1:100 in 1% BSA/PBS and incubated for two hours at room temperature. This was followed by incubation with the secondary antibody, donkey anti mouse IgG Alexa 594 (Cat.No.: 715-585-151, Jackson ImmunoResearch, Cambridge, United Kingdom), diluted 1:500 in 1% BSA/PBS for one hour at room temperature. Cells were washed with PBS containing 0,02% Tween-20 (Cat.No.:9127.1, Carl Roth). DNA was counterstained with DAPI (1 g mL^−1^, Cat.No.: D9542, Sigma-Aldrich Chemie GmbH (Merck)) for 10 min.

### MTT viability assay

Media was removed from the chamber followed by adding 50 µL of serum-free DMEM and 50 µL of 3-(4,5-Dimethyl-2-thiazolyl)-2,5-diphenyl-2H-tetrazolium bromide (MTT, Cat.No.: M2128, Sigma-Aldrich Chemie GmbH (Merck)) solution into the chamber and incubating at 37 °C for 3 h. After incubation, the MTT solution was removed and 100 µl 2-propanol (Cat.No.: 131090.1212, Th.Geyer GmbH und Co.KG) was added into each chamber and incubated for 15 min. Subsequently, solution was transferred into a 96-well plate and absorption at 570 nm was measured (TECAN, SPARK® multimode microplate reader, Männedorf, Switzerland).

### Microscopy

To analyze the uptake of molecules into the cells as well as the cell’s viability, cell cycle and genotoxicity we used a Leica laser scanning confocal microscopy. For full MW chamber multi-image stitching, we used a Nikon spinning disk confocal microscope. All details on the systems are found in Table S3.

### Image analysis and statistics

All software details are given in Table S4.

For cell survival, counting cells and creating montages of confocal microscopy images was performed using Fiji (https://magej.net/software/fiji/). The mean cell counts were obtained from counting the cells from at least 10 microscope images or performing image stitch over 12 frames per experiment using the cell counter plugin (cell_counter.jar). Subsequently, the survival rate (%) was calculated from the respective data with respect to the mean of the control.

For cellular uptake, the subcellular distribution of the particular substance was scored and the cells showing the expected subcellular localization of the substance counted as above and the percentage from the total cells calculated.

For cell cycle analysis, we scored the % of cells in mitosis using the contrast image and morphological analysis of the cells. Cells in mitosis depict a round morphology which is distinguishable from the interphase cells.

Statistical significance (p-value) was calculated using a two-sided t-test.

### Simulations

The simulations of the electrical field strengths as well as the temperature simulations of the MWP device were done using CST MWS Suite 2023 from Dassault Système (Table S4). The MWP device was designed using the materials described above in “Design and performance of the microwave porator device”. The simulations were performed at an input power of 25 dBm and the resulting electrical field strengths in the MWP device chamber were scaled in dependence of the simulated S-parameters to match the electrical field strengths inside the fabricated MWP device.

## Results

### Performance of the microwave porator device

**Fig. 2:**
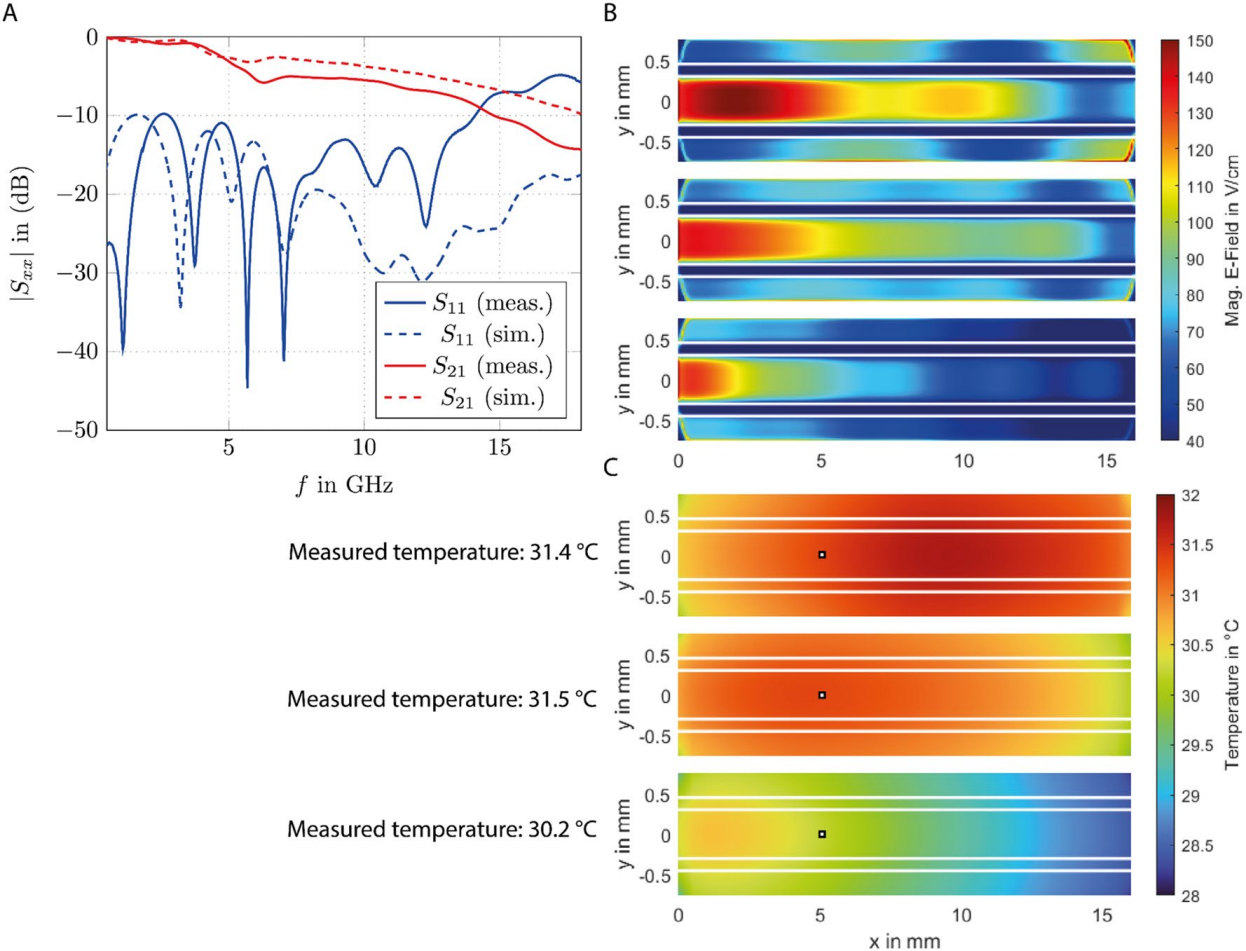
Performance of the microwave porator device. (**A**) The S-parameters of the MWP device for the simulated and the fabricated MWP device. The S-parameters are ratios of the incident and reflected or the transmitted and reflected microwave, showing the frequency operation range of the device (S_11_ < − 10 dB). For high frequencies (13–18 GHz) the differences between the simulated and fabricated MWP device are high and had to be compensated using higher input power levels. (**B**) Image of the simulated electrical field strengths for 5.8 GHz (top image), 10 GHz (middle image) and 18 GHz (lower image) at 25 dBm along the CPW (marked with white lines). (**C**) Simulated steady-state temperature distribution along the CPW (marked with white lines) at 25 dBm for 5.8 GHz (top image), 10 GHz (middle image) and 18 GHz (lower image). The spots where the temperature was measured in the MWP device are marked by a rectangle. The measured temperature is given on the left side of the image.

To analyze the performance of the MWP device with the performance obtained from simulation, the ratio of the incident to reflected wave at the input port of the device (S_11_) and the ratio of the transmitted to reflected wave at the output port (S_21_) of the simulated and measured MWP device were compared with each other. The S-parameters are a measure of how much power is effectively fed into the device (S_11_) and how much of this power is transmitted through the device (S_21_). Figure 2A shows the S-parameter results for S_11_ and S_21_ in a frequency range from 0.5 to 18 GHz. In the frequency range from 0.5 to 14 GHz the ratio of the incident wave to the reflected wave S_11_ is <  − 10 dB for the measured MWP device. For a frequency range of 14–18 GHz the highest S_11_ ≈  − 6 dB is reached. In general, for a frequency range of 0.5 and 8 GHz there is a shift in the S_11_ in frequency between the measured and simulated S_11_. For higher frequencies the amplitude of S_11_ differs. This can be explained by a not optimal soldering connection between the SubMiniature version A (SMA) connector and the MWP device as well by the influence of the used biocompatible glue to mount the rexolite container to the glass. A comparison of the simulated and measured S_21_ showed that there was a small difference in the simulation model and the fabricated device, resulting in less power transmitted during the experiments than in the simulation model. Using the measured S-parameters and power levels measured by a power meter, the simulated power levels were modified such that the simulated power matches the measured one. To do this, the differences between the measured and simulated S-parameters were computed and the resulting factor was used to match the simulation results to the one in the device. Due to this, the simulated electrical field strengths were also matched to the one in the fabricated MWP device. In Fig. 2B the simulated electrical field strength along the glass bottom of the chamber for different frequencies (5.8 GHz, 10 GHz and 18 GHz) at 25 dBm is shown. The power level is chosen to reach high electrical field strengths while at the same time avoiding high temperatures. In general, the electrical field strength along the trace of the CPW (y =  − 0.3 mm to y = 0.3 mm) decreased much faster for 18 GHz (bottom image) compared to 5.8 GHz (top image). Interestingly, along the gap of the CPW (marked with white lines, y = 0.3 mm to y = 0.45 mm) the electrical field strength was minimal while the maximal electrical field strength values were reached along the trace, starting at the side of the chamber where the microwave entered the chamber. Furthermore, the electrical field strength reached high levels at the rim areas of the chamber, especially at 5.8 GHz and 10 GHz. Varying the power level of the incident wave allowed us to analyze the influence of the electrical field strength on the cell membrane permeabilization along the chamber by comparing the simulated electrical field strength with the microscope images of the cells taken after the MWP experiments. The maximal electrical field strength reached along the chamber was 150 V cm^−1^ at an input power of 25 dBm and 5.8 GHz.

Next, we performed temperature simulations at an input power of 25 dBm, as well as temperature measurements. The power for the temperature simulation had been compensated in comparison to the fabricated device to ensure that the simulated temperatures were comparable to the temperature measurements. Figure 2C shows the simulated temperature distribution for 5.8 GHz, 10 GHz and 18 GHz in the steady-state case for an input power of 0.31 W (25 dBm). Due to the higher reflections, the resulting maximal temperature at 18 GHz is 31 °C, while a temperature of 32 °C is reached at 5.8 GHz. Interesting is the shift of the hot spot in the chamber: at 5.8 GHz the hot spot is at 1/2 to 2/3 of the length of the chamber, at 10 GHz it is at 1/3 of the length of the chamber and at 18 GHz it is at 1/4 of the length of the chamber. The measured (marked by rectangles in Fig. 2C) and simulated temperatures matched well, in temperature value as well as in temperature distribution. Furthermore, due to the maximal temperature of only 32 °C whereas optimal mammalian cell cultivation temperature is 37 °C, a temperature-only effect on the cell membrane and with this on the uptake process itself can be excluded. These temperature-only effects have been also excluded in our previous work^5^.

### Effect of microwave treatment on cell survival, cell division cycle and genotoxicity

**Fig. 3:**
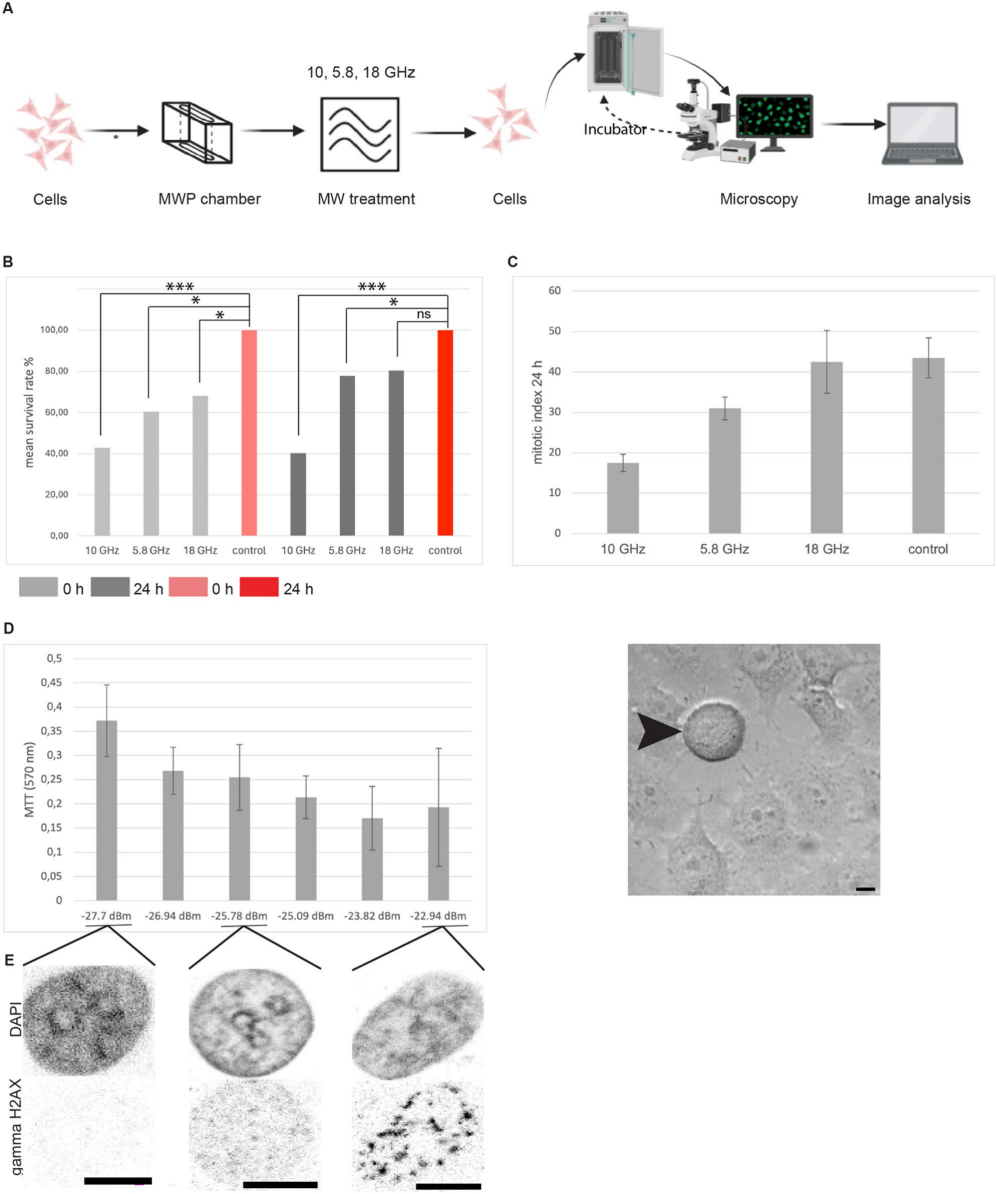
Effect of microwave (MW) treatment on cell survival, cell cycle and genotoxicity. (**A**) Graphical summary of the experiments. Human HeLa cells were seeded on the MWP chamber and the next day, cells were subjected to MW treatment as indicated. Immediately after or the next day, cells were analyzed by microscopy for their survival relative to control unirradiated cells, their proliferation ability by scoring the mitotic index or assayed for cell viability using the MTT assay. See “Methods” for details. (**B**) Survival rate (%) was calculated by determining the mean cell count for all experiments, separated in untreated control 10 min (N = 4) and microwave treatment at 10, 5.8 and 18 GHz for 10 min (N = 4), with an electrical field strength from 30 to 150 V/cm. The p-value was determined using the t-test (see “Methods”). ns > 0.05; *p < 0.05; **p < 0.01; ***p < 0.001. (**C**) To score for effects on cell cycle and cellular proliferation, the cells undergoing cell division (mitotic index) were counted one day after the MW irradiation. Values are given as mean ± SD. The image below, depicts one mitotic cell (round morphology, arrowhead) surrounded by several cells in interphase (flat morphology). (**D**) Cell viability was assessed by the MTT assay at 10 GHz with increasing electrical field strength. Data shown are representative of N = 3 independent biological experiments and values are given as mean ± SD. (**E**) To test for genotoxic effects of MW irradiation, cells were stained with an antibody to the phosphorylated form of histone variant H2AX (γ-H2AX) after treatment with 10 GHz at different electrical field strength as indicated. To further visualize the genomic DNA, cells were counterstained with the DNA specific dye DAPI. The punctate staining over the DAPI labeled DNA, indicates DNA double strand breaks. Scale bars = 10 μm.

Next, we investigated whether microwave treatment had an effect on cell survival, cell cycle, and/or genotoxicity. To test that, we made use of human cells growing as adherent cultures. This represents the majority of cell types in humans with exception of blood cell types, which naturally grow in suspension cultures. The graphical representation of the experimental strategy is shown in Fig. 3A.

To assess the short and long term effects on cell survival we counted the cells upon microwave treatment at different frequencies immediately following treatment as well as after 24 h (Fig. 3B). Control samples were also included under the same conditions but in the absence of microwave irradiation. shown in Fig. 3B there was a difference in the survival of HeLa cells at 18 GHz and 5.8 GHz compared to 10 GHz. The mean survival rate at 10 GHz compared to the control was around 60% lower, while differences in the range of 20–40% were observed at both 18 GHz and 5.8 GHz, respectively. This result clearly indicates that the viability of cells at 10 GHz is strongly reduced. Furthermore, there were only minor differences in cell survival between immediately after microwave treatment and one day later.

We then analyzed if the cell division cycle is influenced by the different frequencies. For that purpose, the mitotic index, i.e., the number of cells dividing, was determined after 24 h (Fig. 3C; mitotic cell depicted by an arrowhead). We found that the mitotic index of cells treated at 18 GHz was not affected relative to control unirradiated cells. On the other hand, the mitotic index decreased at 5.8 GHz and even more at 10 GHz. Hence, the irradiation at 18 GHz, showed the least effect on both cell survival and cell division and at 10 GHz, likely cell death and/or cell detachment occurs.

Hence, we next analyzed cell viability at 10 GHz with increasing electrical field strength, using a MTT assay (Fig. 3D). The MTT assay was conducted after 24 h. The data showed a clear decrease in viability with increasing electric field strength.

To further investigate potential toxic effects of treatment of cells with 10 GHz, we determined genotoxic effects using an immunostaining with gamma H2AX. This antibody labels regions within the genome that suffered DNA double strand breaks^18^. These are the most severe types of DNA damage and, if unrepaired, lead often to cell death. The results depicted in the Fig. 3E showed a clear increase in the numbers and the size of the gamma H2AX labeled foci, indicating the occurrence of DNA double strand breaks by irradiation at 10 GHz and depending on the electrical field strength.

Altogether, the decreased cell survival, cell division and cell viability combined with an increased level of genotoxic effects at 10 GHz, makes this condition not recommended for cell transfection using microwaves. Furthermore, our data indicate that treatment at 18 GHz has the least toxic effects for the cells. Efficient microwave-induced cellular uptake of small substances is possible at 18 GHz.

### Cellular uptake of substances up to whole antibodies

**Fig. 4:**
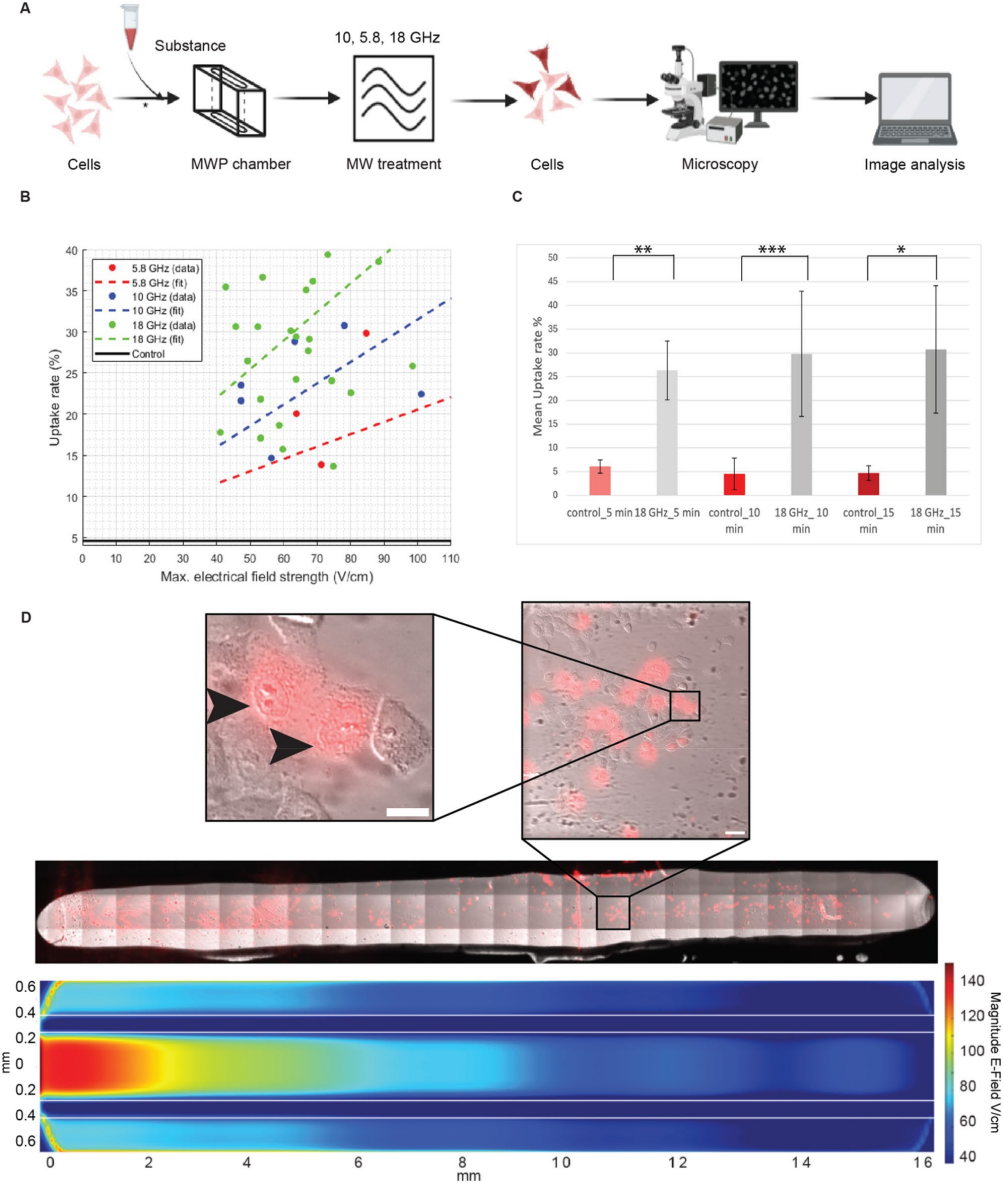
Microwave-induced cellular uptake of small substances. (**A**) Graphical summary of the experiments. Human HeLa cells were seeded on the MWP chamber and the next day, cells were subjected to MW treatment as indicated. At the times indicated, cells were analyzed by microscopy for the MW-induced intracellular uptake of the cell-impermeable dye propidium iodide (PI) and compared with the uptake in control unirradiated cells. (**B**) Uptake rates of PI in HeLa cells over maximum electrical field strength in the device at different frequencies and 10 min MW treatment. Each dot represents one experiment in a chamber filled with around 5000 cells. For every experiment 10 randomly acquired images along the CPW were chosen and the total number of cells in the images as well as the number of positive cells (cells that took up the substance PI) were counted. With these values, the uptake rate in % was computed. The electrical field strength is the maximal electrical field strength along the CPW in the experiment. (**C**) Mean uptake rate (%) at 18 GHz for three different times after MW irradiation. This was calculated by counting the percentage of cells (from all cells in the fields of view) labeled with PI in control samples without MW treatment, at 5 min (N = 3), 10 min (N = 3) and 15 min (N = 3) and in MW treated cells after 5 min (N = 4) 10 min (N = 9) and15 minutes (N = 3). Values are given as mean ± SD. N = number of independent biological replicates. The p-value was determined using the t-test (see “Methods”). *p < 0.05; **p < 0.01; ***p < 0.001. (**D**) Stitched microscope images showing PI uptake by the cells over the whole MWP device at 18 GHz. The uptake is correlated to the electrical field strength distribution shown in the bottom. LUT is shown at the side. For better visualization of the uptake of PI by the cells and labeling of the DNA and RNA inside the cells (arrowheads), parts of the images are enlarged above the stitched image. Scale bars = 10 μm.

To explore the effectiveness of microwave-induced cellular uptake of small substances, the experimental approach depicted in Fig. 4A was conducted using the nucleic acid dye propidium iodide (PI). With these experiments, the enhanced permeability of HeLa cells was verified through the uptake of propidium iodide. Typically, propidium iodide cannot penetrate intact membranes; however, when the cell membrane is compromised, the propidium cation (Pr2+) can traverse the membrane and interact with the nucleic acids inside the cell, leading to subsequent fluorescence^19^. Furthermore, we aimed to determine the extent to which the frequency and other parameters affect the cellular uptake. We assessed the impact of frequency, along with various electrical field strengths, using the frequencies of 5.8 GHz, 10 GHz, and 18 GHz as depicted in Fig. 4B. It is evident that the cellular uptake rates in experiments conducted at 18 GHz are clearly higher compared to those at 5.8 GHz and 10 GHz. Notably, the electrical field strengths, typically in the V cm^−1^ range rather than the kV cm^−1^ range seen in conventional electroporation, remain consistent across all frequencies analyzed. In summary, we conclude that both, high survival rates and the high cellular uptake rates, can be obtained with the frequency of 18 GHz. This condition was also successful using our initial MWP device prototype^5^.

To test whether longer treatment times lead to higher cellular uptake, we scored PI uptake at 5, 10 and 15 min of MW treatment. The outcome shown in Fig. 4C depicts only minor differences when extending the time indicating that most uptake of PI can be already observed at 5–10 min post MW.

Because the electrical field is not homogenous along the chamber (Fig. 2B), we examined the cellular uptake over the entire chamber at 18 GHz (Fig. 4D). For that purpose, we acquired images covering the whole chamber and stitched together. Along the entire length of the MWP device, cells remained predominantly attached. For better visualization of the cell morphology and PI uptake (PI inside cells is seen as red color overlaid with the contrast image of the cells), different magnifications of sections of the chamber are shown above. A large number of cells is seen with PI labeling the DNA and RNA in the cell. Comparing these experimental observations with simulations of electrical field strength (see Fig. 4D, bottom), a discernible cellular uptake pattern emerges, indicating a correlation between electrical field distribution and uptake rate. However, no threshold voltage is visibly evident that would initiate or trigger the uptake process. Certain areas, particularly within the hot spot and regions of high electric field strength in the MWP device, exhibited elevated uptake rates. Generally, a higher uptake rate is observed along the trace of the CPW compared to the ground area of the MWP device. These results reveal a relation between the uptake rate R_Up_, the electrical field strength E and the power P which can be described by

$${\text{R}}_{{{\text{Up}}}} \sim {\text{P}} \sim {\text{E}}^{{2}} .$$

**Fig. 5:**
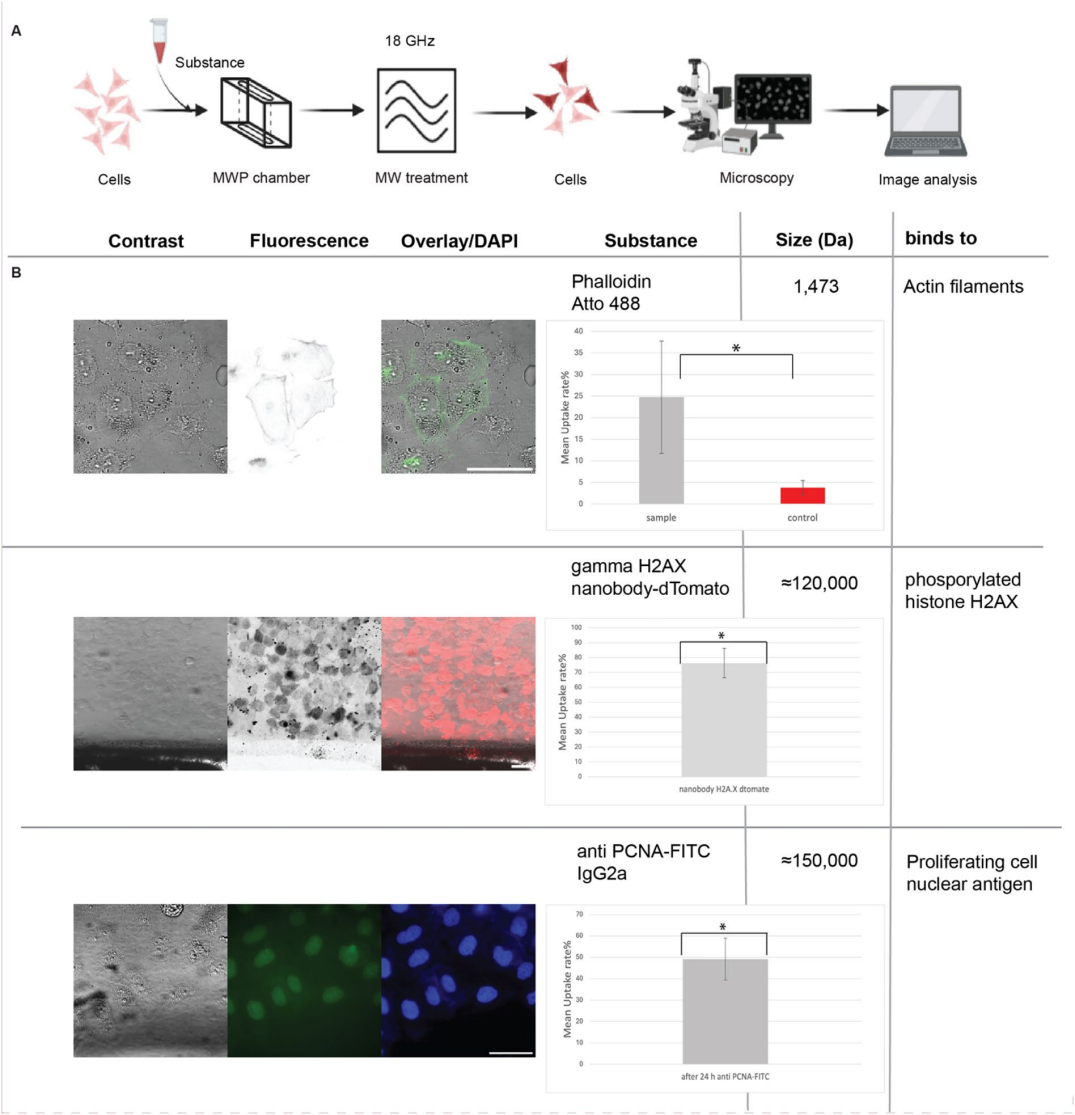
Microwave-induced cellular uptake of different size substances. (**A**) Graphical summary of the experiments. (**B**) To test uptake of substances of different chemical composition and size, human HeLa cells were treated with MW at 18 GHz for 10–15 min while being incubated with a series of membrane impermeable substances including: Atto 488-conjugated phalloidin (label of the cells depicts actin cytoskeleton in green over contrast image), two γ -H2AX-specific nanobodies conjugated with a tandem dTomato fluorescent protein and a nuclear localization signal (nuclear histone H2AX signal shown in red over contrast image), and an entire immunoglobulin G antibody to PCNA conjugated with FITC (signal shown in green and nuclear DNA counterstained with DAPI). On the left hand side, a contrast image of the cells is shown to highlight all cells, this is followed in the middle column by the fluorescence signal of the substance indicated and at the right hand side the overlay of both images depicting the cells that took up the substance over the contrast image of all cells. For PCNA uptake the nuclear DNA is shown instead of the contrast image, Additionally, the mean uptake rate % was calculated for all three samples, for phalloidin with microwave treatment (N = 6) and without microwave (N = 4) and for the nanobody/antibody (N = 3). The p-value was determined using the t-test (see “Methods”). Also, the substance’s name, size (in Dalton) and the intracellular binding targets of the substance are given. Scale bars = 50 μm.

Having ascertained the best conditions for MW poration of small chemical substances (PI) with the lowest cell toxicity, we next tested using the same conditions whether other non-cell permeable substances covering a large range of sizes and chemical properties, as well as endogenous target sites inside cells (Fig. 5A). We selected Phalloidin, fused to Atto 488; a natural bicyclic peptide with a size of 1473 Da that binds highly selective to actin cytoskeleton filaments in the cell’s cytoplasm. As shown in green in the second row in Fig. 5B, a very clear label of the cytoskeleton fibers inside the cells is visible resulting from the uptake and the binding of the phalloidin conjugated with the green emitting Atto 488 fluorophore to filamentous actin. We were also able to extend these data to another cell type and mammalian species, in particular, we successfully performed uptake of both PI and phalloidin in mouse myoblast cells (Fig. S1). Finally, we selected two different types of antibodies. One was a camelid-derived nanobody selected to bind gamma H2AX. This construct contains two copies of the anti-gamma H2AX nanobody VHH genetically fused to a dimeric fluorescent protein dTomato, emitting red fluorescence. The primary antibody was an immunoglobulin G (IgG) raised against PCNA, conjugated with FITC and emitting green fluorescence. The sizes of the conjugates were on the order of 120,000–150,000 Da, and the Y-shaped tetrameric IgG dimensions are 14.5 nm × 8.5 nm × 4.0 nm. After MW treatment in the presence of anti-PCNA IgG, we further incubated the cells over two days to allow cell division and access of the antibodies into the cell nucleus as these do not contain a nuclear localization signal. As shown in the lower two rows in Fig. 5B, multiple cells are labeled with either of the antibodies, indicating that microwave treatment at 18 GHz allowed for transient cell permeabilization and uptake of large substances including protein quaternary complexes such as IgGs. As before, we quantified the mean percentage uptake for the three substances and obtained uptake values between 25% for Phalloidin and up to 80% for the nanobody. Importantly, cell morphology was unaltered, in agreement with our results above (Fig. 3).

## Discussion

**Fig. 6:**
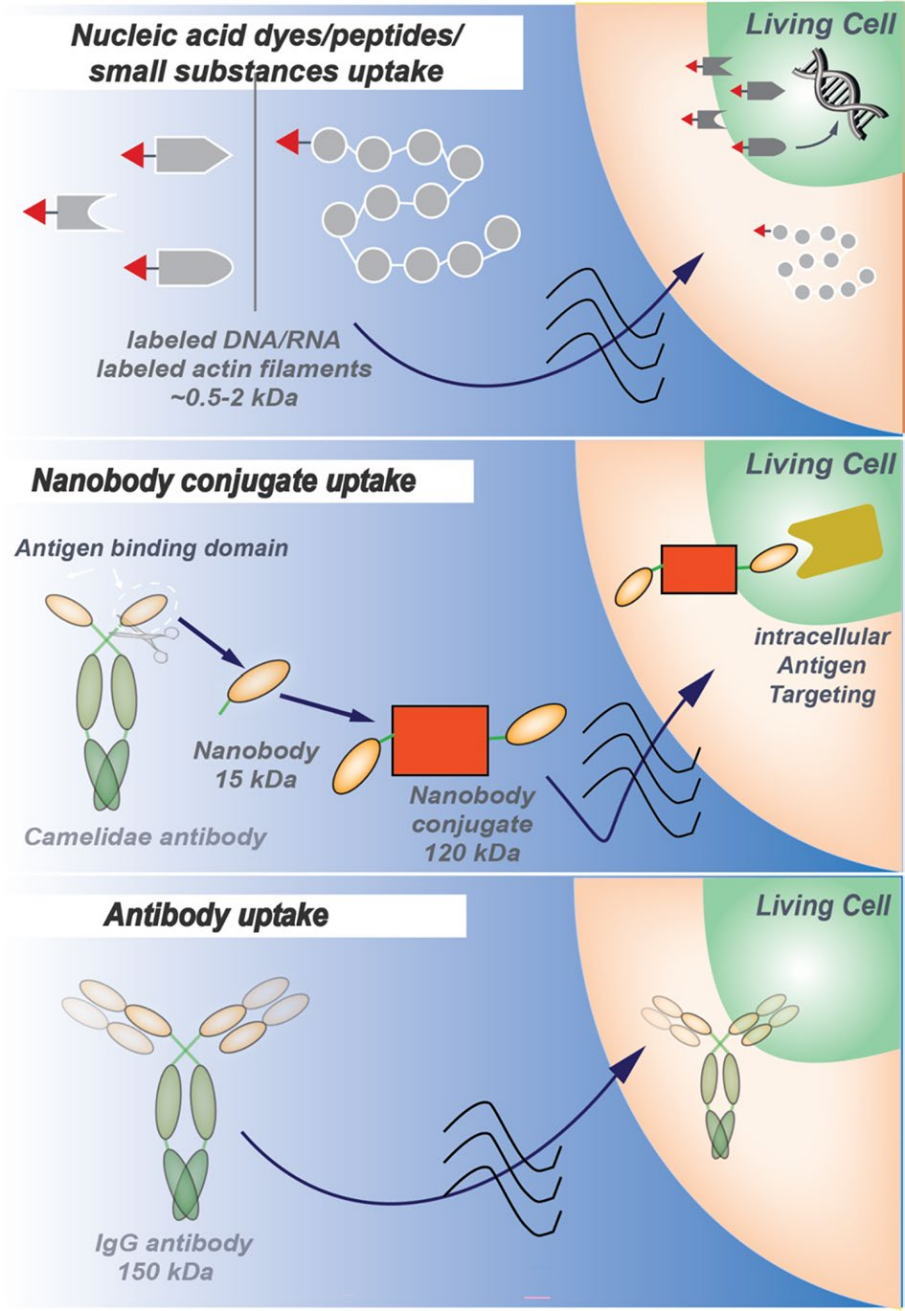
Graphical summary of microwave induced uptake of substances of different sizes and composition into cells.

We were able to demonstrate uptake of various substances with different molecular composition and sizes in human and in mouse cells (Fig. 6). These substances include small chemical dyes which bind and label nucleic acids inside the cytoplasm and nucleus of the cells, a substance labeling actin filaments in the cytoplasm of the cells as well as large protein conjugates which detect histone modifications within the cell’s nucleus. Hence, we could show delivery of molecules and their localization at all major subcellular compartments and binding to different targets inside the cells. Importantly, and in contrast with conventional electroporation methods, this technique does not require the cells to be detached from the substrate before MW treatment. Cell detachment not only temporarily drastically changes cell physiology but also interrupts the observation until the cells can attach again, which normally takes several hours. So microwave treatment of adherent mammalian cells in the 18 GHz range offers a promising avenue for studying cell (path)physiology in life sciences, both from a basic research point of view and for potential biomedical applications.

Insights into the cellular uptake mechanism could lead to new approaches in medicine, for example, by enabling targeted delivery of drugs to specific cell types. By improving performance and increasing throughput, the improved chamber type described here enables more efficient investigation of cells and their responses to various substances. Although the exact mechanisms of substance uptake is not fully understood, a few mechanisms have been proposed in the literature. Li et al.^20^ has shown for a frequency of 10 MHz that the membrane becomes permeable, but there is only a small window of reversible electroporation. As the experiments shown here were performed in a frequency range from 5.8 to 18 GHz, the electrical field strengths are much lower, which results in not reaching these high membrane potentials, because most of the power of the microwave is absorbed by the surrounding media (buffer) and the cytoplasm of the cell. Due to this, the death rates of the microwave treated cells are lower compared to experiments in the radio wave frequency range (MHz) and classical electroporation, which indicates that a more gentle effect occurs, which is less harmful to the cells. An effect which has been proposed to occur at low electrical field strengths is electro-endocytosis^21,22^, where low electrical field strengths force an endocytic uptake mechanism of substances into living cells. These effects, though, were shown to occur at low electrical field strengths at low frequencies, not in the GHz range as used in our study. In our studies we could not observe any evidence of endocytic vesicle formation, and no indication for endocytosis to play a role in the uptake of substances under our conditions. Our conclusion is also in agreement with a previous report using human tumor cells and stating that there is no endocytic uptake in the GHz range as uptake of the substance used occurred in the presence of endocytosis inhibitors^6^. The molecular dynamics simulations of Tharuschi Perera et al.^14^ at 18 GHz with a very high field strength using artificial lipid vesicles to mimic cellular membranes and silica nanospheres as the substance to be delivered indicated changes in membrane lipid area, hydrophobic core (dis)order, membrane thickness, cation presence and water dynamics. Although most of these effects were attained at higher electric fields than the ones used in our study, the authors propose that, at lower electric fields, vibrations and reorientation of water molecule dipoles was sufficient to induce membrane permeability without changing the integrity of the artificial lipid vesicles. Albeit this model was obtained from simulations with artificial lipid vesicles and it does not take into consideration the complexity of the in vivo situation, it proposes an attractive mechanism explaining the uptake of molecules into cells using MW irradiation without compromising cell viability. Altogether, the experimental data shown here indicate that there is a relation between the uptake rate R_Up_, the electrical field strength E and the power P. The frequency f seems to be a main parameter for an increased cellular uptake rate R_Up_, because the uptake rate at 18 GHz is much higher compared to 10 GHz or 5.8 GHz at similar electrical field strengths. This could be related to the resonance frequency of bound water molecules at f_res_ ≈ 18 GHz as proposed by Tharuschi Perera et al.^14^. In conclusion, this technology holds great promise for life sciences research and applications as it allows intracellular delivery of a variety of substances into cells with low impact onto cell physiology.

## Supplementary Information

Supplementary Information.

## Data Availability

All the data can be found here: https://tudatalib.ulb.tu-darmstadt.de/handle/tudatalib/4191.
