# Supplementary material for: Uptake of substances into living mammalian cells by microwave induced perturbation of the plasma membrane
Source: Sci Rep. 2024 Sep 6;14:20885. doi: 10.1038/s41598-024-71401-7 (PMC11379910; doi:10.1038/s41598-024-71401-7)
Supplement: Supplementary file 1 — Supplementary Information. [file 41598_2024_71401_MOESM1_ESM.docx]

# Uptake of substances into living mammalian cells by microwave induced perturbation of the plasma membrane

# *Manuela Milden-Appel^1,§^, Markus Paravicini^2,§,^*, Jannick P. Milden^1^, Martin Schüßler^2^, Rolf Jakoby^2^*, *M. Cristina Cardoso^1,^**

# *^1^* Cell Biology and Epigenetics, Department of Biology, Technical University of Darmstadt, Germany

# *^2^* Institute of Microwave Engineering and Photonics, Technical University of Darmstadt, Germany

^§^ First authors

* Correspondence to: M. Cristina Cardoso ([cardoso@bio.tu-darmstadt.de](mailto:cardoso@bio.tu-darmstadt.de)) and Markus Paravicini ([markus.paravicini@tu-darmstadt.de](mailto:markus.paravicini@tu-darmstadt.de))

# Supplementary Figures

**
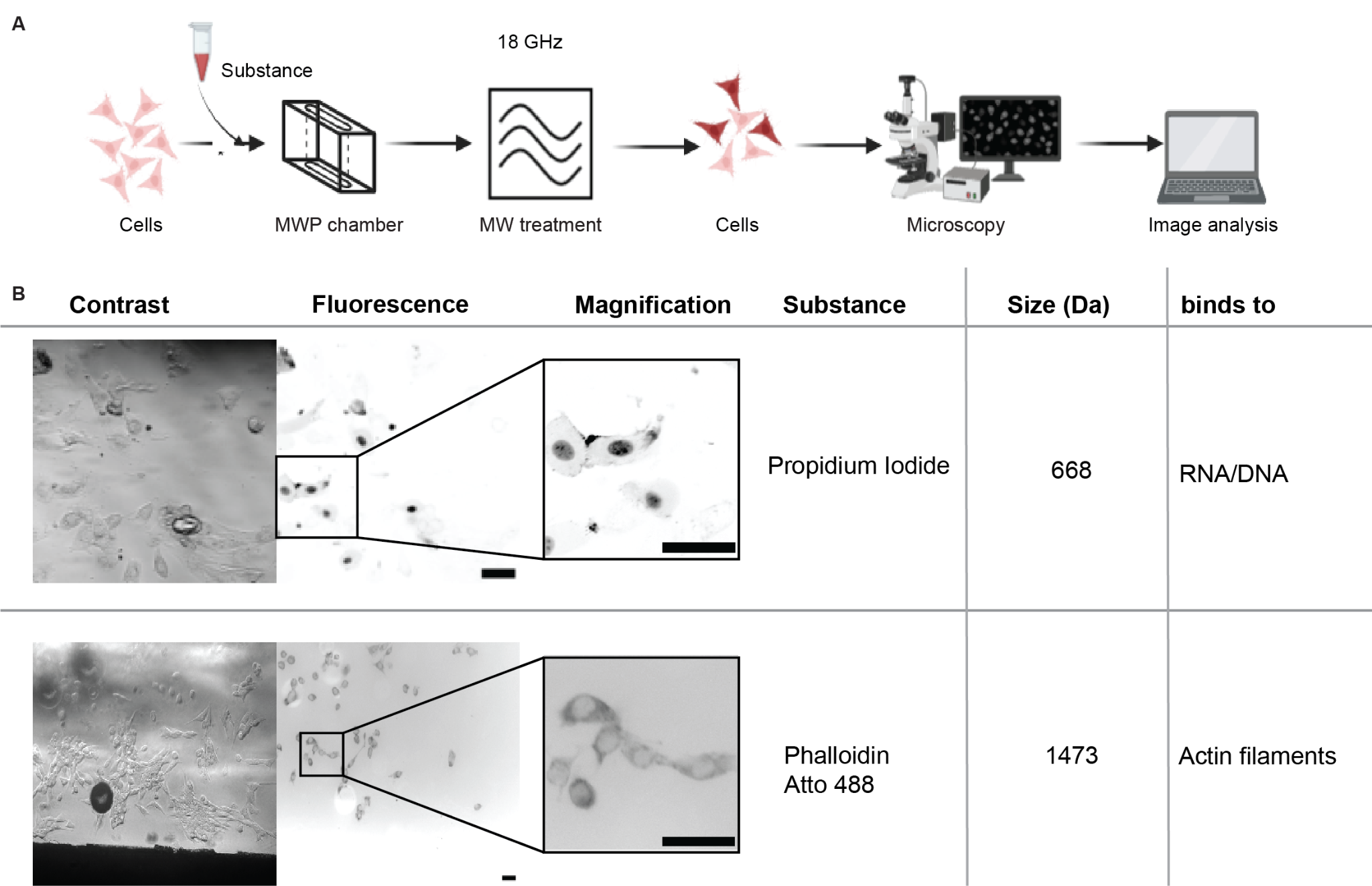
**

**Figure S1. Microwave-induced cellular uptake of different size substances in mouse cells.** A) Graphical summary of the experiments. B) To test uptake of substances of different chemical composition and size, mouse C2C12 myoblast cells were treated with MW at 18 GHz for 10-15 minutes while being incubated with a series of membrane impermeable substances including: propidium iodide (which labels DNA and RNA inside cells) and Atto 488-conjugated phalloidin (label of the cells depicts actin cytoskeleton). On the left hand side, a contrast image of the cells is shown to highlight all cells followed in the middle column by the fluorescence signal of the substance indicated and at the right hand side a magnified region is shown to better depict the cells that took up the substance and the different intracellular distribution related to their intracellular binding sites. In addition, the substance’s name, size (in Dalton) and the intracellular binding targets of the substance are given. Scale bars = 50 μm.

# Supplementary Tables

# Table S1. List of substances.

| **Substance** | **Size (Da)** | **Concentration** | **Cat. No** | **Company/Reference** |
| --- | --- | --- | --- | --- |
| Propidium Iodide | 668 | 150 µM | P4170 | Biomol GmbH,  Hamburg, Germany |
| Phalloidin-Atto488 | 1,473 | 6.6 µM | A412379 | ThermoFisher Scientific, Waltham,MA,USA |
| gamma H2AX nanobody-dtomato  (C6B-dTo) | ≈120,000 | 5 µM | - | [^22^](https://sciwheel.com/work/citation?ids=11395312&pre=&suf=&sa=0) |
| mouse monoclonal IgG_2a_ PCNA antibody clone PC-10 labeled with FITC | ≈150,000 | 8 µM | sc56-FITC | Santa Cruz Biotechnology, Inc. Dallas, Texas, USA |

**Table S2.** Measurement and power devices.

| **Device** | **Model** | **Company** | **Application** |
| --- | --- | --- | --- |
| RF Amplifier | BLMA 0818 20 | BONN Elektronik GmbH, Germany | Power amplification of microwave |
| RF source | MG3692A | Anritsu K.K., Japan | Power source for microwave generation |
| Power meter | NRV 828.2511.02 | Rohde & Schwarz | Power level monitoring |
| Thermometer | 2116 | Eurotherm, United Kingdom | Thermal monitoring |
| Thermometer | OEM-PLUS | Weidmann Technologies, Germany | Thermal monitoring |

**Table S3.** Imaging systems.

| **Device** | **Light Sources** | **Filters**  **(ex & em [nm])*** | **Objectives/ Lenses** | **Detection system** | **Application** |
| --- | --- | --- | --- | --- | --- |
| PerkinElmer Life Sciences Ultra-View VoX spinning disc on an inverted Nikon Ti-E microscope | Solid state diode lasers (405 nm, 488 nm, 561 nm, 640 nm) | 405/488/561**  ex.405: em.415–475  ex.488: em.505–549  ex.561: em. 580–650 | SPlan Fluor 20x LWD DIC air NA 0.7  2.3 mm | cooled 14-bit Hamamatsu ® C9100-50 EMCCD | cellular viability, cell cycle and cellular uptake of substances |
| Leica TCS SPEII | Solid state lasers (405 nm, 488 nm, 561 nm) | ex.360/40  em.425 LP  ex..470/40  em.515 LP  ex.546/10  em.585/40 | HC PL Fluotar 20x NA 0.5 air 1.15 nm  ACS APO 63x NA 1.3 oil 0.16 nm | single trans- and a single epi-PMT detector | cellular viability, cell cycle, genotoxicity and cellular uptake of substances |

*ex: excitation and em: emission, ** dichroic mirror specification, NA: numerical aperture, PMT: photomultiplier tube, EMCCD: electron-multiplying charge coupled device camera

**Table S4.** Software.

| **Name** | **Version** | **Website** | **Company/University** | **Application** |
| --- | --- | --- | --- | --- |
| CST Studio Suite | 2024 | https://www.3ds.com | Dassault Système, France | EM and thermal simulations |
| MATLAB | R2023b | https://de.mathworks.com | The MathWorks Inc, USA | Data analysis and plotting |
| ImageJ | 1.53c | <https://imagej.nih.gov/ij/> | Wayne Rasband, National Institutes of Health, USA | Image processing and image analysis |
| Adobe Illustrator | 28 | <https://www.adobe.com/> | Adobe, USA | Graphical sketch and figures arrangement |
